# Variation in Performance and Resistance to Parasitism of *Plutella xylostella* Populations

**DOI:** 10.3390/insects10090293

**Published:** 2019-09-11

**Authors:** Rieta Gols, Gaylord A. Desurmont, Jeffrey A. Harvey

**Affiliations:** 1 Laboratory of Entomology, Wageningen University & Research, Droevendaalsesteeg 1, 6708 PB Wageningen, The Netherlands; 2 EBCL USDA ARS, 810 Avenue du Campus Agropolis, 34980 Montferrier-sur-Lez, France; gdesurmont@ars-ebcl.org; 3 Department of Terrestrial Ecology, Netherlands Institute of Ecology, Droevendaalsesteeg 10, 6708 PB Wageningen, The Netherlands; j.harvey@nioo.knaw.nl; 4 Department of Ecological Science, Section Animal Ecology, VU University Amsterdam, De Boelelaan 1085, 1081 HV Amsterdam, The Netherlands

**Keywords:** diamondback moth, insect-herbivore interactions, genetic variation, host-plant adaptation, host immunity, parasitoid, plant resistance

## Abstract

Two major ecological factors determine the fitness of an insect herbivore: the ability to overcome plant resistance strategies (bottom-up effects) and the ability to avoid or resist attack by natural enemies such as predators and parasitoids (top-down effects). In response to differences in selection pressure, variation may exist in host-plant adaptation and immunity against parasitism among populations of an insect herbivore. We investigated the variation in larval performance of six different *Plutella xylostella* populations originating from four continents when feeding on a native Dutch plant species, *Brassica rapa.* One of the used populations has successfully switched its host plant, and is now adapted to pea. In addition, we determined the resistance to attack by the endoparasitoid *Diadegma semiclausum* originating from the Netherlands (where it is also native) and measured parasitoid performance as a proxy for host resistance against parasitism. Pupal mortality, immature development times, and adult biomass of *P. xylostella* differed significantly across populations when feeding on the same host plant species. In addition, parasitism success differed in terms of parasitoid adult emergence and their biomass, but not their development times. Variation among natural populations of insects should be considered more when studying interactions between plants and insects up the food chain.

## 1. Introduction

Insects are the most speciose group of organisms on earth. Their small size, the enormous variation in life-history traits, and physiological adaptations they exhibit allow them to adapt to living in virtually every terrestrial and freshwater habitat. Half of the currently described insect species are phytophagous and depend on plants for their development and survival. Phytophagy requires specific adaptations to extract sufficient nutrients from plants that are considered nutritionally imbalanced, especially in the levels of nitrogen and phosphorous, compared to animal-based resources [1,2]. In addition, insect herbivores have to overcome a range of chemical and morphological defensive strategies that plants have evolved to prevent attack by herbivores and other attackers [3]. The short life-cycles of insects in variable environments could result in rapid genetic changes over generations giving rise to high levels of genetic heterogeneity, allowing them to respond rapidly to environmental change [4,5]. Moreover, phenotypic plasticity provides additional means to adapt to environments that change within the life cycle of an insect [6,7].

Two important ecological factors that determine the success of an insect herbivore are the ability to overcome plant resistance strategies (bottom-up effects) and to avoid or resist attack by parasitoids and other natural enemies (top-down effects). To deal with the first problem, most insects feed on a restricted number of plant species or plants within a family and have evolved adaptations to deal with adverse traits in these host plants, that is they are, to variable degrees, food-specialists [6,8,9,10]. Insects also have a primitive immune system that could prevent infection by invading organisms such as parasitoids of which the offspring develops in or on the bodies of other insects [11]. Similar to the co-evolutionary arms race between plants and insects, this phenomenon also exists between insects and parasitoids [11]. Indeed, Price et al. [12] argued that chains consisting of three or even four trophic levels are involved in strong reciprocal evolutionary interactions. As a result of differences in the strength of top-down and bottom-up selection pressures, as well as stochastic processes, variation may exist in host-plant adaptation and immunity against parasitism among and between populations of an insect herbivore [13,14]. 

The now cosmopolitan pest insect herbivore *Plutella xylostella* L. (diamondback moth: Lepidoptera; Plutellidae) is a good example of a species that has been very successful in expanding its original range, most likely the Mediterranean region, to a global distribution, at least in some parts of the year [15]. Larvae of *P. xylostella* are adapted to feed on glucosinolate-containing plant species (Brassicales), primarily in the Brassicaceae. A sulfatase in the larvae efficiently prevents the formation of toxic glucosinolate-hydrolysis products allowing them to indiscriminately feed on these plants that are toxic to most generalist herbivores [16]. Being a specialized herbivore of vegetable (cabbage) and oilseed (mustard) crops in the Brassicaceae, its range expansion is assisted by the global cultivation of its host plants which has increased by more than 40% from 1990 to 2014 [17]. 

*Plutella xylostella* is known to be migratory [18] resulting in populations that may homogenize over time, and consequently have little genetic structure [19] and express little variation in biological traits. For instance, populations from temperate and tropical regions in Asia displayed little variation in thermal responses [20], which could be attributed to the high mobility of the species. Alternatively, a lack of genetic structure could also be explained by founder effects when this species is introduced inadvertently [19]. Nevertheless, genetic variation in *P. xylostella* populations has been reported with respect to resistance to insecticides suggesting that some differences in dispersal patterns may exist. For example, dispersal of *P. xylostella* is low in agricultural settings where high-quality host plants are available in large numbers [21]. 

In general, agriculture has resulted in a simplification of the landscape. This has affected the interaction between herbivores and their natural enemies in various ways and at different scales [22,23]. The performance of many herbivores, in particular, less well-adapted generalists, has benefitted from a reduction in foliar concentrations of defensive plant allelochemicals in cultivars [24]. Moreover, large stands of monocultures often increase herbivore densities because crop plants are easier to find than wild food plants growing in more structurally complex natural habitats [25,26]. Crop plants and insect herbivores have evolved together since the advent of agriculture several thousands of years ago at the site of domestication. Moreover, global trade has accelerated the occurrence of new interactions between insects and plants. As such, agricultural ecosystems provide excellent systems to study evolutionary change, which could be rapid [27]. In this study, we examine the variation among populations of *P. xylostella* with respect to its performance (survival, development time and adult biomass), on a wild brassicaceous plant species (*Brassica rapa* L.). In addition, we examined resistance to and suitability for parasitism by *Diadegma semiclausum* Hellèn (Hymenoptera; Ichneumonidae), an important larval endoparasitoid of *P. xylostella* [15,28].

The populations of *P. xylostella* used in this study originate from different geographical realms (Western Europe, East Asia, North America, and Africa). In addition, we included a population that has become adapted to feed on sugar pea in Kenya [29]. *Brassica rapa* is native to Western Europe, but its cultivated varieties (e.g., turnip, Chinese cabbage) have a worldwide distribution [30]. We hypothesize that the *P. xylostella* populations, with the exception of the pea-adapted population, exhibit relatively little variation in performance on *B. rapa* because this herbivore is a specialist of cultivated and wild brassicaceous species. However, due to the geographical difference in the community of natural enemies, we hypothesize that there is more variability among the *P. xylostella* populations in host suitability for *D. semiclausum.* For instance, *D. mollipla* is considered the most important parasitoid of *P. xylostella* in Kenya, whereas this is *D. insulare* in the Nearctic [28].

There was significant variation in the performance of the *P. xylostella* populations when reared *B. rapa* host plants in terms of pupal survival, moth biomass, and development time. Differences were most pronounced for moth biomass and lower biomass was not restricted to the pea-adapted population. The various host populations also differentially affected survival and biomass of Dutch *D. semiclausum*.

## 2. Materials and Methods

### 2.1. Insects and Plants

Diamondback moths originated from different laboratories to which we hereafter refer to as populations. The Dutch population was obtained in 2013 from caterpillars found on cabbage plants growing in the vicinity of Wageningen and has been reared for >10 generations on Brussels sprout plants (*Brassica oleracea* var. gemmifera cv. Cyrus). The population from Japan was kindly provided by Dr. Kaori Shiojiri, Kyoto University, Japan. This population was collected from cabbage fields near Kyoto and is replenished one or twice a year. In the laboratory it was been reared on a *B. rapa* cultivar (var. perviridis). Two populations originated from Kenya. One was collected from infested pea fields in Naivasha in 2000 and replenished during the following two years [29]. The second population was collected from cabbage fields 40 km south of Nairobi. Both populations were sent to the Max Planck Institute for Chemical Ecology in Jena, Germany, in 2005 and were maintained, since then, on their respective food plants (*B. oleracea* and *P. sativum* cultivars) in the laboratory [31]. These two populations were also maintained at the University of Amsterdam in the Netherlands and moths were kindly provided by Dr. P. Roessingh. The population from the US, exact origin unknown to us, was provided by Syngenta, Stein, Switzerland. The French population was collected in France and was provided by the French Institute for Agricultural Research (INRA). The rearing history of the latter two populations is unknown to us.

The larval endoparasitoid *Diadegma semiclausum* was also collected in 2013 in Wageningen from the same location where the moths originated. A culture was maintained on the Wageningen *P. xylostella* population on Brussel sprout plants heavily infested with early instar *P. xylostella* larvae. Adults that emerged from the cages were collected and used for experimental or rearing purposes. Adult parasitoid wasps were >5 days old when used for parasitism (see Experimental Design).

Upon arrival in Wageningen in February 2014, the caterpillars or adult moths were used to generate one generation (F1) on Brussel sprouts. Adult moths, males and females (40–100 in total per population), were released in a cage with a Brussel sprout plant that served as a substrate for oviposition. After 48 h the adults were collected, killed by freezing, and discarded. The eggs and larvae were allowed to develop into adults, and their offspring were used in the bioassays (see below). The *P. xylostella* populations were maintained in a greenhouse at 22 ± 3 °C, 50% relative humidity, and a photo-regime of 16:8 (Light:Dark). If the light intensity dropped below 500 μmol photons m^−2^ s^−1^ light was supplemented using high pressure sodium light (SON-T lights). Various populations were kept in insect cages (45 × 45 × 60 cm) each placed within an insect-proof net to exclude escape of adults and mixing of the various populations. 

Brussel sprout plants were grown in 1.1 L pots filled with potting soil (Lentse Potgrond, # 4, Lent, the Netherlands) and were 5–6 weeks old when fed to the insects. *Brassica rapa* seeds were collected from a natural population growing in the vicinity of Wageningen. Plants were grown from these seeds in 2.5-L pots filled with potting soil. When the plants were four weeks old, they were fertilized once a week using a commercial fertilizer (Kristallon blauw NPK 19-6-20). Plants were 7–8 weeks old when insects were introduced onto the plants (between March 13 and 17, 2014).

### 2.2. Experimental Design

Adult F1 moths were allowed to oviposit on *B. rapa* plants for 24 h. Following oviposition, the adults were collected using an aspirator and transferred to a second cage with a *B. rapa* plant for another 24 h after which they were frozen and discarded. When the caterpillars reached the second larval instar (L2), they were transferred to the experimental *B. rapa* plants using a fine paintbrush. Thirteen caterpillars were transferred to a single plant, and 10–11 plants were used per *P. xylostella* population. A second group of L2 larvae was parasitized by *D. semiclausum* females. Individual *P. xylostella* hosts were presented to a female wasp. A caterpillar was considered to be parasitized when the wasp inserted her ovipositor into the host, retracted it, and moved away from the host. This usually takes between 1 and 2 s. Eleven parasitized hosts were introduced onto single plants, and there were ten plants per *P. xylostella* population. Individual plants were covered with insect nets (100 × 66 cm) supported by wooden sticks to prevent cross-contamination. Insects were allowed to move and feed freely within a net. When all the larvae had pupated or developed into parasitoid cocoons, they were collected and transferred to labeled 5-mL glass vials (one pupa/cocoon per vial). When adult moths or wasps eclosed, their sex and development (egg-to-adult) were recorded, and the wasps were killed by freezing. Adult eclosion was checked every 2 h. Insect dry mass was determined by drying the insects for three days at 80 °C and weighing them on a microbalance (CP2P, Sartorius, Göttingen, Germany). Experiments were conducted in a greenhouse under the same conditions as the insect rearing. 

### 2.3. Statistical Analysis

Development time (egg-to-adult) and adult dry mass of the moths was analyzed using a general mixed model analysis of variance with *P. xylostella* population and sex as fixed factors and plant as a random factor. Data for the male parasitoids were also analyzed using a general mixed model as for the moths but without sex as a main factor. The sex ratio of the laboratory population of *D. semiclausum* was highly male-biased and, as a result of this, the experiments generated relatively few females. Therefore, we used a general linear ANOVA for analyzing development times and adult biomass of the female parasitoids. To reveal sex-related differences, we also analyzed the data for male and female parasitoid together with a general linear model with sex included as a model term. The number of moths/parasitoids that survived until adulthood was analyzed using a generalized linear model with a binomial distribution and a logit link function. Here, the number of eclosing adults of the initially released caterpillars served as the response variable and *P. xylostella* population as the explanatory variable. In addition, we determined whether pupal mortality differed among the *P. xylostella* populations. Here the number of individuals that was recovered from the plant but did not survive to adulthood out of the initially released caterpillars served as the response variable. To investigate whether any of the performance variables (survival, development time, and adult mass) were correlated, we performed Spearman’s correlation tests on the mean values obtained for each population. All analyses were performed using SAS 9.3 [32].

## 3. Results

### 3.1. Survival and Performance of Healthy Moths 

**Figure 1:**
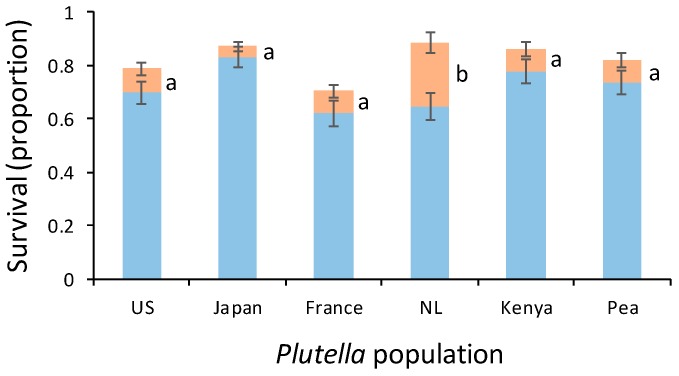
Survival to adulthood (blue bars: mean proportion ± SE) of *Plutella xylostella* caterpillars from populations originating from the US, Japan, France, the Netherlands (NL), and Kenya, as well as a population adapted to pea in Kenya. Orange bars present the proportion of *P. xylostella* pupae that did not emerge as adults. Orange bars with the same letter are not significantly different (blue bars are not significantly different from each other). For each population, 13 caterpillars were introduced on each of 10 to12 *Brassica rapa* plants.

Survival (to adulthood) of the various *P. xylostella* populations on *B. rapa* plants varied between 62 and 83% on average but was not significantly different (χ^2^_5_ = 8.99, *p* = 0.11, Figure 1). However, pupal mortality did differ among the populations (χ^2^_5_ = 25.2, *p* < 0.001) and was significantly higher in the Dutch *P. xylostella* population (Figure 1). 

**Figure 2:**
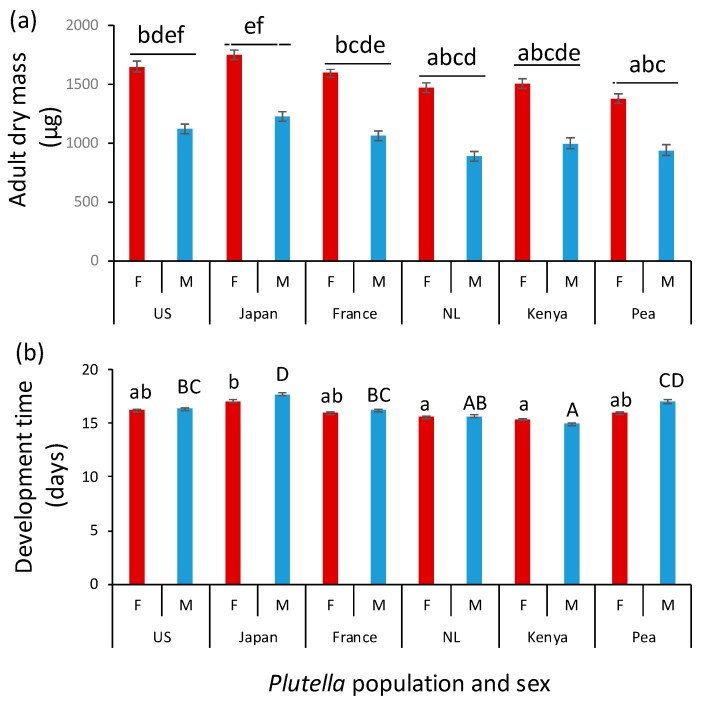
Mean (±SE) adult dry mass (**a**) and egg-to-adult development time (**b**) of females (red bars) and males (blue bars) of *Plutella xylostella* populations originating from the US, Japan, France, the Netherlands (NL), and Kenya, as well as a population adapted to pea in Kenya. Thirteen caterpillars of each population were introduced on each of 10 to 12 *Brassica rapa* plants. Male (capital letters) and female (small letters) data were analyzed separately in panel (**b**). Bars with the same letter are not significantly different.

When reared on *B. rapa*, adult dry mass varied among the populations and differed between males and females (population: F_5, 56.6_ = 11.52; *p* < 0.001; sex: F_1, 497_ = 1075; *p* < 0.001, Figure 2a). Sex-related size-dimorphism was similar in all populations (F_5, 496_ = 1.58; *p* = 0.16); females were approximately 50% heavier than males. The dry mass of the adult moths ranked from low to high: Pea, the Netherlands, Kenya, France, US, Japan. Japanese *P. xylostella* moths were almost 30% larger than the Pea moths.

Egg-to-adult development times differed among the populations (F_5, 56.7_ = 9.49; *p* < 0.001) and between the sexes (F_1, 517_ = 5.4; *p* = 0.02) (Figure 2b). The effect of the interaction between population and sex on development time was also significant (F_5, 517_ = 3.49; *p* = 0.004). With the exception of the Kenyan moths collected from cabbage, females tended to develop faster than males, and this was more pronounced in moths originating from Japan and the Pea-adapted population. The effect of the *P. xylostella* population on development time was more pronounced in males than in females. The difference between the slowest (Japan) and fastest (Kenya) development was 1.7 days (11%) for females and 2.7 days (17%) for male moths. 

### 3.2. Survival and Performance of Parasitized Caterpillars

**Figure 3:**
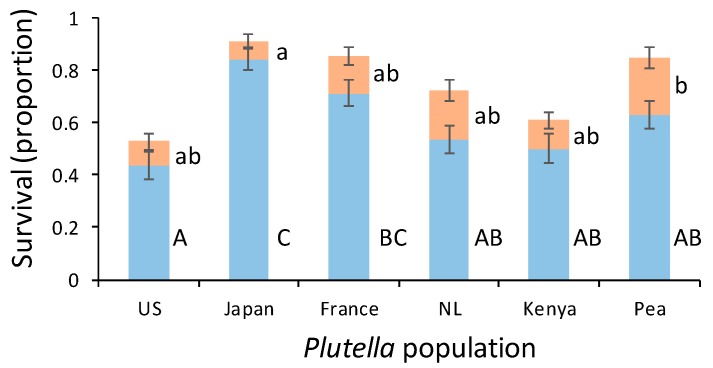
Survival to adulthood (blue bars: mean proportion ± SE) of *Diadegma semiclausum* from *Plutella xylostella* host populations originating from the US, Japan, France, the Netherlands (NL), and Kenya, as well as a population adapted to pea in Kenya. Orange bars present the proportion *D. semiclausum* cocoons that did not emerge as adults. Orange (small letters) and blue bars (capital lettes), respectively, with the same letter are not significantly different. For each population, 11 parasitized caterpillars were introduced on each of 10 *Brassica rapa* plants.

Approximately 5% of the parasitized caterpillars that survived to adulthood produced moths. Eclosion of moths occurred in all *P. xylostella* host populations. When parasitized by *D. semiclausum* originating from the Netherlands, the fraction of successful parasitoid emergence depended on where *P. xylostella* originated (χ^2^_5_ = 40.2, *p* < 0.001, Figure 3). Successful parasitism was significantly lower on US and higher on Japanese hosts. Some of the mortality could be attributed to the population-related pupal mortality (χ^2^_5_ = 13.5, *p* = 0.02, Figure 3). This mortality was significantly higher when parasitizing the pea-adapted population compared to the Japanese population. 

**Figure 4:**
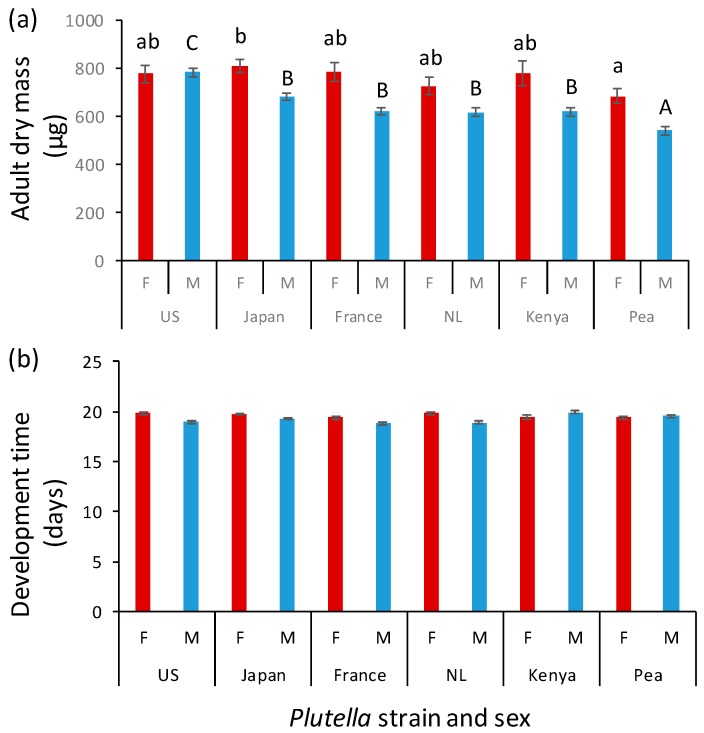
Mean (± SE) adult dry mass (**a**) and egg-to-adult development time (**b**) of female (red bars) and male (blue bars) *Diadegma semiclausum* developing from *Plutella xylostella* populations originating from the US, Japan, France, the Netherlands (NL) and Kenya, as well as a population adapted to pea in Kenya. Eleven parasitized caterpillars of each population were introduced on each of 10 *Brassica rapa* plants. Bars with the same letter are not significantly different. Data were analyzed separately for females (small letters) and males (capital letters).

Adult mass of both males (F_5, 42.4_ = 19.4; *p* < 0.001) and females (F_5, 86_ = 2.42; *p* = 0.04) varied with host origin (Figure 4a). The lightest wasps, both males and females, eclosed from pea-adapted hosts. The largest females developed in Japanese hosts, whereas the largest males developed in US hosts. (Figure 4a). The effect of host population was larger for the lighter sex, i.e., males. The difference in weight between the heaviest and lightest wasps based on population mean values was approximately 45% for males, i.e., male US wasps were on average 45% heavier than male Pea wasps, and 12% for females (Japan vs. Pea). Size dimorphism had disappeared when *D. semiclausum* developed on *P. xylostella* from the US.

Development times on the various host populations were similar for female wasps (F_5,90_ = 0.41, *p* = 0.84), whereas they were more variable for male wasps (F_5,54.6_ = 2.08, *p* = 0.08) (Figure 4b). Males tended to develop faster than females but this was reversed for the Kenyan pea and cabbage population, although this result was not statistically significant (Figure 4b). 

### 3.3. Correlation Analyses

Pair-wise correlation analyses of the performance variables (Table 1) revealed that the adult biomass of males and females of both species were positively correlated. In addition, the biomass of the parasitoids correlated positively with the biomass of unparasitized caterpillars. This result suggests that the sexes respond similarly to plant nutritional quality, and more importantly, that if the food plant is of high quality for the hosts, it is also of high quality for the parasitoids, at least in terms of biomass. Interestingly secondary fitness correlates, i.e., biomass and development time, did not correlate with the primary correlate, survival. Neither did the survival of *D. semiclausum* correlate with that of unparasitized hosts. The development time of male *D. semiclausum* correlated positively with the survival of unparasitized *P. xylostella*. This means that on populations with high survival of healthy hosts, *D. semiclausum* males took longer to develop, which seems a bit of an anomaly. 

## 4. Discussion

*Plutella xylostella* originating from four different continents and six populations expressed differences in host-plant performance and suitability to parasitism by a Dutch *D. semiclausum* population. Herbivore pupal survival was variable, with particularly high pupal mortality in Dutch *P. xylostella*, whereas parasitoid survival was significantly lower when reared from the American than from the Japanese and French host populations of *P. xylostella*. In terms of adult dry body mass, moths with a recent rearing history on pea were significantly smaller than conspecifics originating from Japan, which was the population with the best performance both in terms of survival and the biomass of the moth and the parasitoid. In general, the biomass of the moths of a specific population correlated with the biomass of *D. semiclausum* developing in hosts from that population. The differences in the development times of the *P. xylostella* populations were more pronounced in males than in females, whereas the development times were quite similar for the parasitoid when developing on the different host populations. 

We had anticipated that all of the *P. xylostella* populations, with the exception of the African one, that had recently adapted to pea, would exhibit little variation in development when reared on *B. rapa*, because this herbivore is a specialist of cultivated and wild brassicaceous species [33,34]. However, deviation in fitness trait expression was not restricted to the pea-adapted population. Given their adaptation to brassicas, we expected that there would have been considerable convergence in selection pressures from plants, leading to limited divergence in the expression of traits such as development. In fact, we expected traits to vary more in accordance with abiotic factors in the countries of origin, such as temperature, as climatic conditions in the different countries vary enormously. They include cool and seasonal temperate (the Netherlands), warm temperate (France, the US), humid temperate (Japan), and tropical (Africa). However, Shirai [20] found that there was little variation in egg production and larval development time among populations collected in temperate and tropical regions in Asia when measured at different temperatures. A possible explanation for this result is the high mobility of this species resulting in homogenization of populations over relatively large geographical ranges [20]. 

Interestingly, the survival of the moths did not correlate with size (biomass) or development time, nor did size correlate with development time. These results suggest that factors either intrinsically (related to the population) and extrinsically (related to the nutritional quality of the food plant) independently determine whether the larvae survive, how fast they grow, and how well they convert food into body tissues. Life-history theory predicts a trade-off between development time and adult size [35,36]. In insect herbivores that feed on leaf tissues, exposure to threats from the environment (e.g., risk of predation/parasitism, dislocation due to rainfall) may prioritize selection for fast development. In a previous study, it was also shown that variation in development time was small and variation in size was large when *P. xylostella* was reared on wild and cultivated brassicaceous species differing dramatically in glucosinolate concentrations [37,38]. Population-related differences in growth rate and food assimilation may consequently have determined differences in the sizes of the moths. Note here the considerable difference in biomass between male and female moths [39]. The performance variables of the parasitoid also correlated poorly. The only positive correlation was found between the mass of unparasitized *P. xylostella* and the mass of adult parasitoids. Biomass of *D. semiclausum* is clearly determined by the growth potential of the host. This parasitoid species has a tissue feeding strategy and, in contrast to parasitoids with a hemolymph-feeding strategy, needs to consume the host completely before it can pupate [39,40], which further explains the congruity between host and parasitoid biomass.

The variation in survival of the parasitoid *D. semiclausum* on the different host populations was significantly larger than the variation in survival among unparasitized hosts. In host insects, hemocytes play an important role in the immune response against invaders such as pathogens and parasitoids, resulting in encapsulation of, e.g., parasitoid eggs [11,41]. *Diadegma semiclausum* and related endoparasitoids in the Campopleginae utilize symbiotic polydnaviruses to overcome host immunity and regulate development [42,43]. We found that *P. xylostella* from the US and Kenya (cabbage) were poor hosts for Dutch *D. semiclausum*: 50% or less of the initially parasitized hosts eclosed as adults compared to more than 80% adult eclosion on Japanese hosts. Differences in host quality for parasitoid development may occur when *P. xylostella* and *D. semiclausum* originate from different geographical regions as a result of physiological isolation, and this may explain these variable parasitism success ratios. In the US, the most common *Diadegma* species attacking *P. xylostella* caterpillars is *D. insulare*, and in east Africa, it is *D. mollipla* [28]. *Diadegma semiclausum* from Taiwan has been successfully introduced in Kenya [44], but this was after the host shift of *P. xylostella* and collection of the herbivores used in this study. Interestingly the survival of the Dutch *D. semiclausum* on Dutch *P. xylostella* was also relatively low. Over the past 20 years in the Netherlands, there has been a shift in larval parasitism of *P. xylostella* from two species (*D. semiclausum* and to a lesser extent *D. fenestrale*) to four species, (*D. fenestrale*, followed by *D. semiclausum, Cotesia vestalis* and *Dolichogenidea sicaria*; personal observations R. Gols and J. Harvey). Geographical genetic variation in host immunity and parasitoid virulence has been reported before among interactions between *Drosophila* hosts and *Leptopilina* pararasitoids [13,45,46]. Differences in local reciprocal selection pressure and or differences in local selection pressure by the community of parasitoids may result in a variation in the immunity and parasitoid virulence and merits further investigation.

In the Netherlands, wild *B. rapa* is an early successional plant that flowers in April, and thus, may not be an important host plant for *P. xylostella*. However, cultivated varieties of *B. rapa* are grown throughout the growing season in many parts of the world including Europe, Asia and North America, although not in Africa [30], and are used as food plants by *P. xylostella*. The local distribution of wild and cultivated brassicaceous plant species, which are both used as host plants [33] and the change in distribution over the growing season may select for variation in host plant adaptation.

Given that *P. xylostella* is a specialist of brassicaceous plants, the switch to pea is noteworthy, considering that secondary (defensive) metabolites from plants in the mustard and pea families differ significantly [47,48]. This switch occurred when densities of the moth were extremely high on cabbage plants adjacent to pea fields [31]. Importantly, although immature survival was quite high, the adult body mass of the pea-adapted *P. xylostella* population was relatively low, suggesting some metabolic costs associated with the host shift reducing its ability to exploit *B. rapa*. One of the costs may be immune resistance to parasitism [11], explaining high parasitoid survival on the pea-feeding population. Another cost of the switch from pea to mustard is that the herbivore is less effective at utilizing and allocating plant tissue to body mass, which is similarly reflected in parasitoid development [49]. Henniges-Janssen et al. [50] investigated the genetic basis for this host-shift and revealed a complex, autosomal oligogenic inheritance. They also found that the alleles associated with pea-adaptation were not yet fixed in the pea population and that survival on pea did not correlate with survival on kale [50]. This result corroborates with our findings on survival. Roßbach et al. [51] investigated the ability of *D. semiclausum* (originating in Taiwan) to improve its performance on *P. xylostella* adapted to pea when reared on this host for three successive generations. They found that pupal mortality, but not larval mortality was higher on pea-adapted hosts and that this did not change in successive generations. Moreover, pupal development was extended, and adult *D. semiclausum* were smaller when reared on pea-adapted compared to cabbage-adapted *P. xylostella* hosts [51]. These fitness variables also did not improve in three successive generations. These results suggest that, even for *D. semiclausum,* there are fitness costs associated with this food plant shift that are not easily overcome [51]. 

Previous studies have investigated genetic differentiation in populations of *P. xylostella* from different geographical origins using genetic markers [19,52,53]. These studies revealed high levels of genetic differentiation between and among populations, but poor genetic structure by distance. The proposed causes for the lack of genetic structure are pesticide-induced mutagenesis, massive migration ability, and commercial cabbage activities [52]. Ultimately, a major aim has been to link diversity based on genetic markers to variation in trait expression. Here, we showed subtle differences in life-history traits among populations originating from four different continents when reared under the same conditions. However, our results should be interpreted with caution as most of the populations have been reared in the laboratory for many years. It cannot be excluded that differences in performance are also the result of rearing *P. xylostella* for many years under laboratory conditions. In plant breeding, host plant resistance is often studied from the plant perspective, i.e., various accessions of a plant species are screened for potential resistance genes using a single lab population of the pest species. This approach ignores the fact that there may also be considerable within-species variation in the expression of life-history traits in the pest. The results of this study show that the variation among populations of insects could be considerable and should be considered more often in studies on plant-insect multitrophic interactions.

## 5. Conclusions

We found that *P. xylostella* originating from four different continents expressed subtle but significant variation in host-plant performance and suitability to parasitism to a Dutch population of *D. semiclausum*. In contrast with our hypothesis, the lesser performance was not restricted to the pea-adapted population and performance was also variable among the populations adapted to cabbage. Performance variables, survival, adult biomass, and immature development time correlated poorly. This suggests that factors affecting survival, duration of immature development, and adult biomass are not necessarily the same. Variation among natural populations and laboratory strains of insects should be appreciated more when studying interactions between plants and insects up the food chain.

## Figures and Tables

**Table 1:** Spearman’s correlation coefficients on performance variables (survival, biomass, and development time) of the six *Plutella xylostella* populations when they were unparasitized (*P. xyl*) or parasitized by *Diadegma semiclausum* (*D. sem*).

Variables	*P. xyl* Sur ^1^	*D. sem* Bio ^2^_F ^3^	*D.sem* Bio_M ^4^	*P. xyl* Bio_F	*P. xyl* Bio_M	*D. sem* Dt ^5^_F	*D. sem* Dt_M	*P. xyl* Dt_F	*P. xyl* Dt_M
*D. sem*–sur	0.14	0.43	−0.09	0.20	0.26	−0.43	−0.20	0.43	0.54
*P. xyl*–sur		0.14	0.09	0.26	0.37	0.09	0.83 *	0.14	0.37
*D. sem* Bio_F			0.83 *	0.94 **	0.89 *	−0.09	−0.31	0.71	0.31
*D. sem* Bio_M				0.94 **	0.89 *	0.20	−0.26	0.71	0.26
*P. xyl* Bio_F					0.94 **	0.14	−0.20	0.77	0.37
*P. xyl* Bio_M						−0.09	−0.03	0.83 *	0.54
*D. sem* Dt_F							−0.09	0.03	−0.09
*D. sem* Dt_M								−0.31	0.03
*P. xyl* Dt_F									0.83 *

^1^ Survival, ^2^ Adult biomass, ^3^ Females, ^4^ Males, ^5^ Development time, * *p* < 0.05, ** *p* < 0.01, highlighted in grey.
